# Forecasting mean deviation in glaucoma patients using an irregular autoregressive time series method

**DOI:** 10.1038/s41598-025-30870-0

**Published:** 2025-12-19

**Authors:** Carlyn Childress, Stuart K. Gardiner, Manoj Pathak

**Affiliations:** 1 Murray State University, Murray, KY 42071 USA; 2 Devers Eye Institute, Legacy Health, 1225 NE 2nd Ave, Portland, OR USA

**Keywords:** Visual field, Mean deviation, Ordinary least square regression, Irregular autoregressive, Computational biology and bioinformatics, Diseases, Medical research, Risk factors

## Abstract

Accurate forecasting of disease progression is vital in glaucoma management. Ordinary least square regression (OLSR) analyses are not appropriate to perform trend analysis on longitudinally collected perimetry data. This study examines the applicability of an irregular autoregressive of order 1 (IAR (1)) method to model mean deviation (MD) series and investigates if IAR (1) improves validity of the model and results better forecasts then OLSR. Longitudinal data from eyes with progressive glaucoma were used. A total of 1200 MD data from forty-two eyes were included in this study. MD series from the eyes were fitted using both OLSR and an IAR (1) methods. A correlogram was used to determine if errors of the fitted OLSR and IAR (1) were correlated. Predictability of the IAR (1) method was then compared with OLSR using forecast Mean Square Error (MSE). Residuals from the OLSR were correlated and did not satisfy the assumption of normality. On the other hand, the IAR (1) model markedly improved the validity of the model as evidenced by insignificant autocorrelation functions (*p*-value > 0.05) and model’s ability to fit heavy-tailed distribution. Compared to the OLSR fit, significantly higher percentages of eyes resulted smaller MSE (62% vs. 38%, *P* = 0.02) when fitted with IAR (1) method. The IAR (1) method adequately addresses the shortcomings of OLSR when fitting repeatedly collected perimetry data. The IAR (1) method appears to be statistically more valid method for fitting MD series and more accurately forecasts MD progression when compared with OLSR fit.

## Introduction

Glaucoma is a leading cause of irreversible blindness worldwide which is characterized by progressive optic neuropathy and damage to the visual field (VF)^1^. Early detection and assessment of glaucoma progression is vital in glaucoma management. The assessment of glaucoma progression is carried out by monitoring functional and structural changes^2–4^ Although both functional and structural changes can provide evidence of disease progression, tracking glaucomatous visual field progression is of key importance because functional testing directly relates to the activities of daily living^5^. Standard automated perimetry (SAP) is currently the most widely used test for detecting functional damage.

Several Studies done in the past have suggested a variety of statistical techniques to predict VF decay in glaucoma patients. Among various methods, trend analyses are increasingly being performed on longitudinally collected perimetry data, such as trend analysis of MD series, VF index and pointwise VF sensitivity data. McNaught et al.^6^, recommended to use polynomial model for fitting and predicting VF data. Caprioli et al.^7^, explored the VF progression using linear, quadratic and nonlinear exponential model. Recently, more complex methodologies have been proposed. Pathak et al.^8–10^, have proposed hierarchical nonlinear mixed effect models to account group effects of hierarchically structured ophthalmic data (both within eye and between fellow eyes of the same subject) as well as temporal correlation of within eye data nested within subject.

Despite the availability of better statistical methods for monitoring functional progression, trend analyses using OLSR are still the most commonly used statistical method to model VF data from an individual eye. Many studies have used clinical applications of OLSR for measuring treatment effects^11^, assessing the rate of VF progression^12^ and examining characteristics of types of glaucoma^13^. The OLSR, despite being the most commonly used method for performing trend analysis, however has been proven to be an inappropriate method for modeling longitudinal data^9,10^. The OLSR are statistical techniques developed to model association between a response and one or several predictors. The OLSR model assumes that the true relationship between the response and a predictor is linear, and model’s errors are uncorrelated, normally distributed and homoscedastic. Pathak et al. in their studies have shown that trend analysis of VF data using OLSR method is insufficient for longitudinal data as errors from OLSLR fit appear to be correlated and heteroscedastic. As a consequence, OLSR method overestimates the significance of trends, giving potentially inaccurate information about disease progression. Furthermore, the OLSR methods are designed to make prediction within the range of predictor (i.e., time in this study) used in the sample data. Extrapolating OLSR to make predictions outside the predictor space often produces unreliable predictions, especially when data are temporally correlated.

Various time series models are available to analyze sequential data collected over time such as Autoregressive Moving Average (ARMA). One of the most commonly used time series methods from the family of ARMA model is an autoregressive method of order 1 or AR (1). These regular time series models assume that observations are regularly spaced in time and are stationary. For a stationary series, certain statistical properties such as mean, variance and autocorrelation structure are all constant over time. Moreover, model errors are assumed to be uncorrelated and normally distributed. The AR (1) and other regular time series methods are also not suitable to fit VF data either, partly because patients’ office visits are not equally-spaced and partly because errors may not be normally distributed. The IAR (1), an extension of AR(1), is a statistical model designed to fit stationary time series data measured irregularly in time. The IAR (1) can be used to both Gaussian data or data with heavy-tailed distribution like Student’s t-distribution. Thus, the objective of this study is to explore if IAR (1) offers a more valid method over OLSR to fit and forecast progression of VF data.

## Methods

### Data

Data from participants with suspected/early glaucoma or with high-risk ocular hypertension from the ongoing Portland Progression Project at Devers Eye Institute in Portland, Oregon, USA were used in this study. The study protocol was approved by the Legacy Health Institutional Review Board. This study complies with the Health Insurance Portability and Accountability Act (HIPPA) of 1996 and is in agreement with the provisions of the Declaration of Helsinki. Consent was obtained from all participants after they were well informed about the risks and benefits of participation.

At baseline, participants either had early glaucoma (SAP MD no worse than − 6 dB) or had ocular hypertension (untreated intraocular pressure repeatedly > 22 mmHg) plus one or more risk factors for developing glaucoma as determined by their eye care provider. Risk factors included age > 70^4,14^, African ancestry^14^ systemic hypertension^15^, peripheral vasospasm^16^, migraine^17^, self-reported family history of glaucoma^18^, disc hemorrhage^19,20^, diet-controlled diabetes^21^ and/or previously diagnosed glaucomatous optic neuropathy or suspicious optic nerve head appearance (cup-disc ratio asymmetry > 0.2), and neuroretina rim notching or narrowing. Participants having visual acuity worse than 20/40 in either eye or worse than mild glaucoma, cataract or media change at baseline were excluded. Other exclusion criteria included any other disease or use of any medications likely to affect the Visual Field (VF), or having undergone intraocular surgery (except for uncomplicated cataract surgery).

SAP was performed with an HFAII perimeter, using the 24-2 test pattern, a size III white-on-white stimulus, and the SITA Standard algorithm^22,23^. All subjects had previous experience with visual field testing prior to entering the study and most had performed multiple previous tests. Tests with > 33% fixation losses or false negatives, or > 15% false positives, were considered unreliable and excluded.

### Statistical model: IAR (1)

Consider a time series $\left\{ {Y_{{t_{j} }} } \right\},j = 1,2,..,n$, observed irregularly at time $\left\{ {t_{j} } \right\}$, i.e., distance between consecutive times $t_{j} - t_{j - 1}$ is not constant. Then, an IAR (1) is defined as follows:

1 $$Y_{{t_{j} }} = \phi^{{\left( {t_{j} - t_{j - 1} } \right)}} \times Y_{{t_{j - 1} }} + \sigma_{Y} \sqrt {1 - \phi^{{2\left( {t_{j} - t_{j - 1} } \right)}} } \varepsilon_{{t_{j} }}$$

where $\varepsilon_{{t_{j} }}$ is the white noise with zero mean and unit variance. Moreover, for the IAR(1) process, $E\left( {Y_{{t_{j} }} } \right) = 0$, and $Var\left( {Y_{{t_{j} }} } \right) = \sigma_{Y}^{2}$ for all $Y_{{t_{j} }}$ where $E\left( {Y_{{t_{j} }} } \right)$ and $Var\left( {Y_{{t_{j} }} } \right)$ are the mean and the variance of the series respectively. The autocorrelation function (ACF) of the process $\left\{ {Y_{{t_{j} }} } \right\}$ is then given by $Corr\left( {Y_{t,} Y_{s} } \right) = \phi^{t - s} ,t < s$^24,25^. The ACF measures the strength of the linear dependency between time series and lagged version of itself. A lag k ACF is the correlation between values that are k units apart and so on. Plots of ACFs at the various lags, called correlogram, is used to check the nature and strength of the temporal dependency. The $\phi ,0 < \phi < 1$, in Eq. (1) is called an autoregressive parameter, measuring the strength of temporal correlation between $Y_{{t_{j} }}$ and $Y_{{t_{j - 1} }} .$ Like regular time series methods, the IAR (1) is also a statistical methodology to fit stationary data but measured irregularly in time. Additionally, the IAR (1) model described by Eq. (1) is not only limited to Gaussian errors but can also be extended to heavy-tailed distribution such as Student’s t-distribution.

### Statistical analysis and assessment of model’s forecast

All analyses were performed using a combination of RStudio and Python programming languages for statistical computation, data manipulation and data visualization. Initially eyes with 25 or more observations were examined. This is because a longer series are desired for a better estimate of the model parameters resulting more precise forecasts. For each eye, the last five observations were put aside as a test data which were later used to compare predictive quality of the fitted OLSR and IAR (1) models. Remaining observations were used, as a training data, to build forecast models. MD series from the training data were then fitted using OLSR with time (year) as a predictor. Only eyes either progressing significantly over time or exhibiting a significant temporal correlation or both, were included in the final analysis. An eye is called progressing if the rate of deterioration is significantly smaller than zero at a 5% alpha level. MD series from an eye is called temporally correlated if the ACF of the residuals of the fitted model is significantly larger than zero at lag 1 at 5% alpha level. The ACF at lag 1 is the value of sample correlation coefficient between two residuals, adjacent to each other. Detrended MD series were then fitted using IAR (1) model. Accuracies of the forecasts from the fitted OLSR and IAR (1) models were then compared using forecast Mean Square Error (MSE).

## Results

A total of 1200 MD values from 42 two eyes with at least twenty-five visits were included in the final analysis. Table 1 presents the characteristic of the study population. The mean follows up time was 18.32 years ($\pm$ 1.22), mean MD at the first and the last visits were 0.29 dB ($\pm$ 2.77) and − 4.09 dB ($\pm$ 6.03) respectively. Among the 42 eyes included in the final analysis, MD series from 15 eyes were temporally correlated as evidenced by significant sample ACF at lag 1 (Table 2). Out of 15 eyes exhibiting temporal correlation, roughly 67% of the them were also deteriorating significantly as evidenced by 95% confidence interval, not containing zero within it (Table 2).

**Table 1:** Characteristics of the study population.

	Mean ($\pm$ SD)	Range
MD series length (visits)	28.72 ($\pm$ 2.10)	(25, 32)
Follow up duration (year)	18.32 ($\pm$ 1.22)	(14.68, 20.40)
MD at first visit(dB)	0.29 ($\pm$ 2.77)	(− 13.05, 2.84)
MD at last visit (dB)	− 4.09 ($\pm$ 6.03)	(− 26.32, 1.79)

**Table 2:** Sample ACF at lag 1, rate of progression, and the corresponding confidence intervals for the rate.

Eye	ACF at lag 1	Rate of deterioration	95% confidence interval for the rate
1	0.73	− 0.45	(− 0.57, − 0.33)
2	0.12	− 0.31	(− 0.41, − 0.22)
3	0.39	− 0.26	(− 0.35, − 0.16)
4	0.59	− 0.20	(− 0.27, − 0.17)
5	0.51	− 0.01	(− 0.12, 0.09)
6	0.6	− 0.47	(− 0.79, − 0.15)
7	0.73	− 0.63	(− 0.72. − 0.55)
8	0.23	0.14	(0.07, 0.21)
9	0.68	− 0.55	(− 0.68, − 0.42)
10	0.5	0.08	(− 0.07, 0.09)
11	0.55	− 2.20	(− 2.49, − 1.91)
12	0.45	− 0.13	(− 0.19, − 0.06)
13	0.61	1.04	(0.63, 1.45)
14	0.13	− 0.05	(− 0.12, 0.03)
15	0.73	− 1.27	(− 1.65, − 0.89)

As a representative case, MD series from one such eye among 15 eyes exhibiting temporal correlation, was fitted using both OLSR and IAR (1). The forecasts made using the OLSR and IAR (1) models are presented in Table 3 with 95% confidence interval. Both OLSR and the IAR (1) models predicted a gradual deterioration of visual field over time. Compared with the actual values, it appeared that the all forecasts from the IAR (1) were in closer agreement with the actual MD value than OLSR forecasts.

**Table 3:** Forecast using OSLR and IAR (1) model.

Time point	Actual MD value	OLSR		IAR (1)	
		Forecast	95% CI	Forecast	95% CI
17.88	− 13.43	− 15.89	(− 17.18, − 14.59)	− 15.43	(− 18.27, − 12.58)
18.83	− 13.53	− 16.16	(− 17.51, − 14.81)	− 15.22	(− 18.06, − 12.38)
18.89	− 14.44	− 16.45	(− 17.86, − 15.04)	− 15.44	(− 18.31, − 12.58)
19.56	− 14.08	− 16.82	(− 18.30, − 15.33)	− 16.25	(− 19.25, − 13.24)
20.06	− 14.04	− 17.10	(− 18.64, − 15.55)	− 16.02	(− 18.87, − 13.24)

**Fig. 1:**
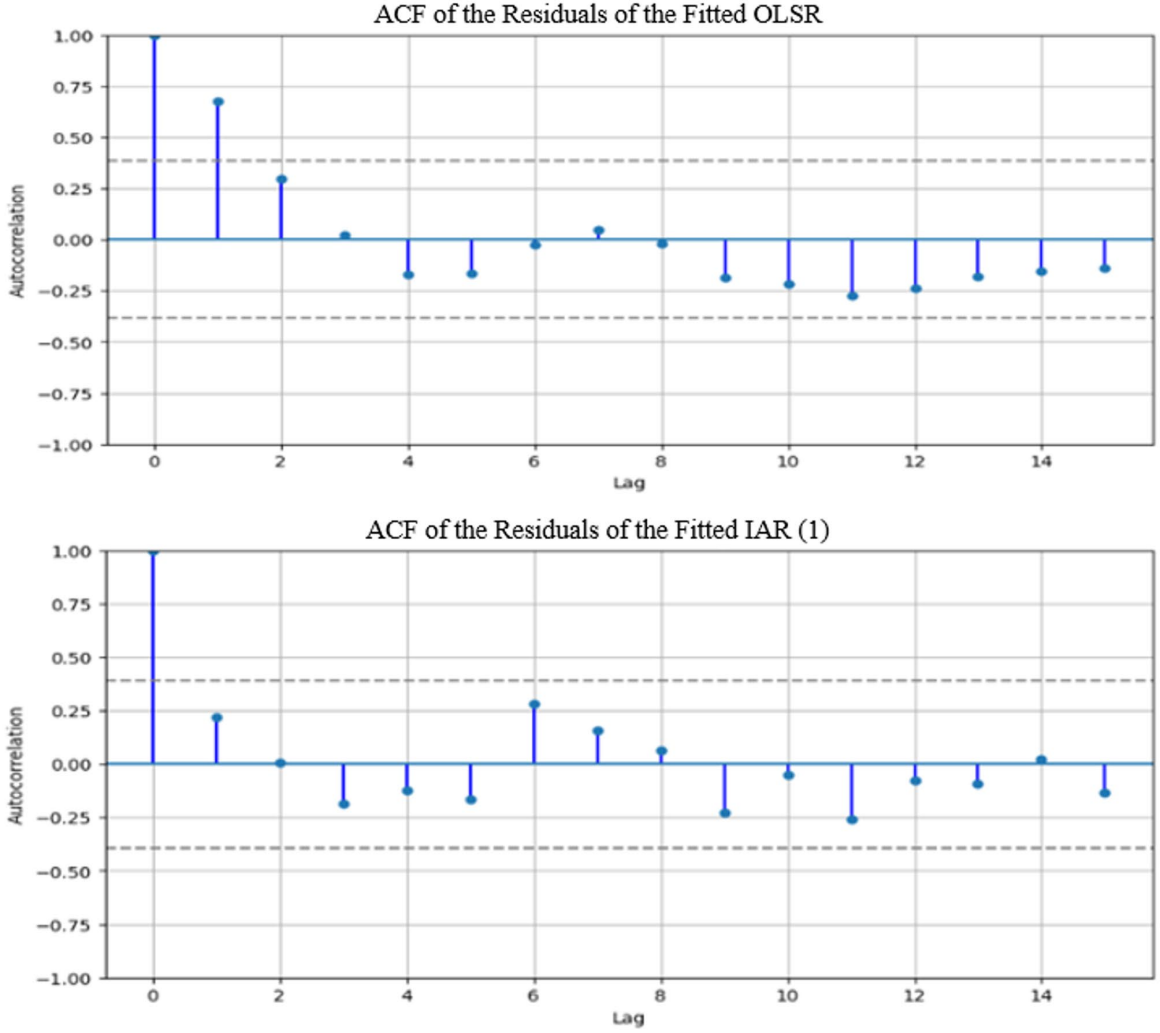
ACF plot of the residuals from the fitted OLSR [top] and IAR (1) [bottom].

Figure 1 displays the ACF plots of the residuals of the fitted OLSR and IAR (1) using the data from the representative eye. It is clear from Fig. 1 that errors of the fitted OLSR were strongly positively correlated at lag 1. This is a clear violation of an OLSR assumption of an uncorrelated error. On the other hand, errors from the IAR (1) fit appeared to be uncorrelated satisfying the IAR (1) model assumption.

**Fig. 2:**
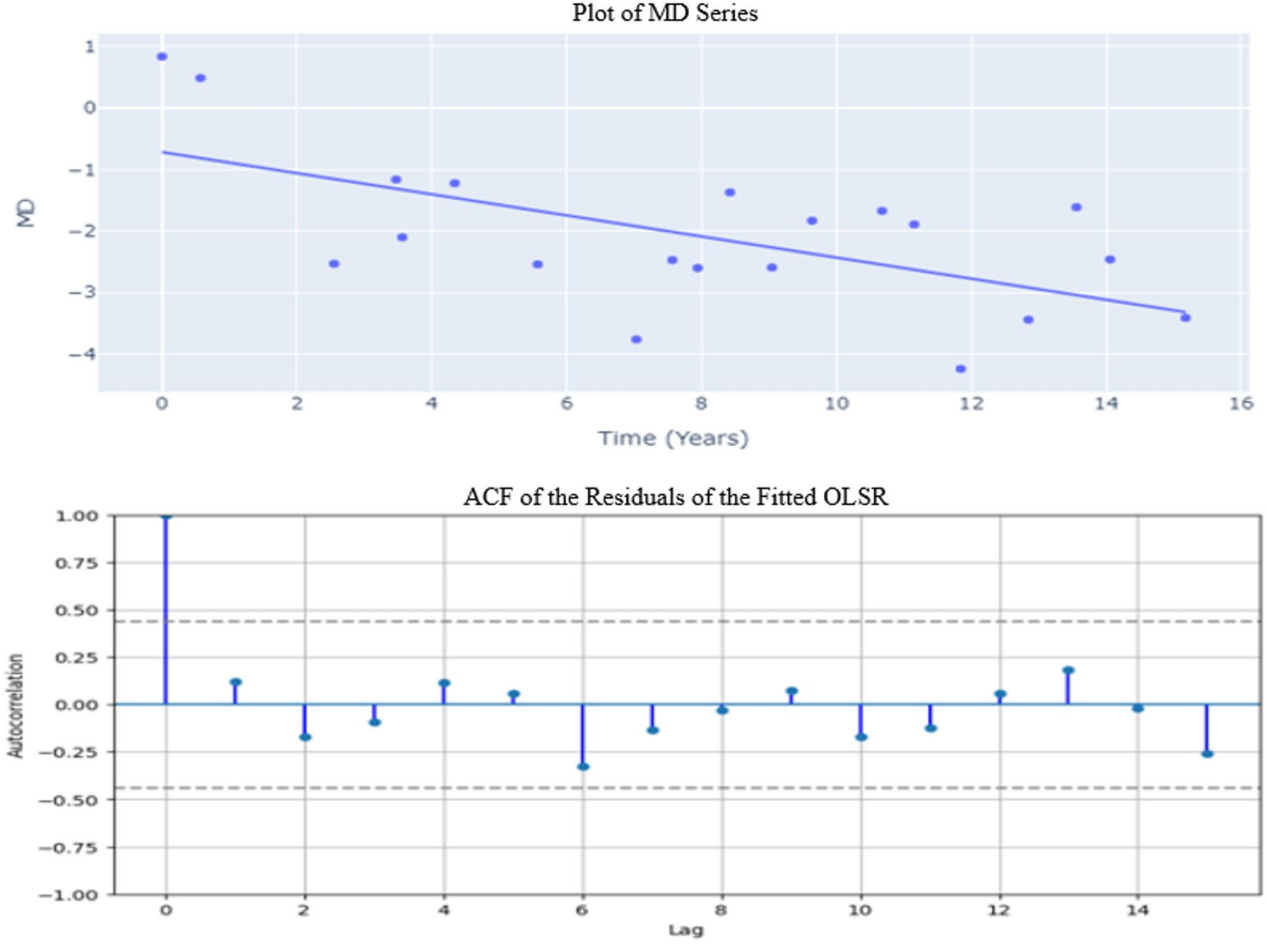
Time series plot (top) and sample autocorrelation of the residuals (bottom) of the fitted OLSR model.

Out of 42 eyes included in this analysis, MD series from the 15 eyes were temporally correlated as discussed above. The remaining 27 eyes showed significant deterioration of the visual field but appeared to be temporally uncorrelated. As another representative case, among those 27 eyes, analysis of MD series from one eye, deteriorated significantly (slope = − 0.1715, *p*-value < 0.05), did not show a sign of temporal correlation as evidenced by non-significant ACF (Fig. 2).

The MD series from the same eye, deteriorating significantly (slope = − 0.1715, *p*-value < 0.05), was then fitted using both OLSR and IAR (1). The forecasts from the fitted OLSR and IAR (1) models are presented in Table 4 with corresponding 95% confidence interval. It appears that the IAR (1) model again resulted a better forecast over OLSR at 4 out of five future time points.

**Table 4:** Forecast of mean deviation from the fitted OLSR and IAR (1).

Time point	Actual MD value	OLSR		IAR (1)	
		Forecast	95% CI	Forecast	95% CI
15.64	− 3.21	− 3.40	(− 4.36, − 2.44)	− 3.47	(− 5.39, − 1.56)
16.16	− 0.70	− 3.49	(− 4.50, − 2.47)	− 3.48	(− 5.41, − 1.55)
16.64	− 1.58	− 3.57	(− 4.63, − 2.51)	− 2.86	(− 4.78, − 0.94)
17.92	− 3.99	− 3.79	(− 4.97, − 2.60)	− 3.73	(− 5.72, − 1.74)
18.40	− 4.62	− 3.87	(− 5.11, − 2.64)	− 3.97	(− 5.90, − 2.04)

Forecast MSEs from the fitted OLSR and IAR (1) methods for all 42 eyes are presented in Table 5. Among 15 eyes with correlated MD series, the IAR (1) method provided more accurate predictions (smaller MSE value) for the majority (12 out of 15) of the eyes. Among 27 eyes that were deteriorated significantly but no sign of temporal correlation, 14 eyes had a smaller MSE, 6 had the same MSE, and 7 eyes had a larger MSE when fitted by IAR(1). . Among all eyes combined, significantly higher percentages of eyes had smaller MSE (62% vs. 38%, *P*=0.02 ) when fitted with IAR (1) method. The IAR (1) outperformed OLSR among eyes with correlated errors as supported by a large reduction in MSE.

**Table 5:** Forecast mean square error from the OLSR and IAR (1).

Eyes with correlated MD series			Eyes with uncorrelated MD series					
	Forecast MSE			Forecast MSE			Forecast MSE	
Eye	OLSR	IAR (1)	Eye	OLSR	IAR (1)	Eye	OLSR	IAR (1)
1	1.34	1.23	16	0.09	0.09	31	0.38	0.51
2	16.8	9.25	17	0.68	0.62	32	1.62	1.17
3	1.29	1.01	18	0.29	0.49	33	0.51	0.51
4	0.15	0.32	19	1.05	1.18	34	0.13	0.13
5	4.64	0.92	20	1.95	1.66	35	2.47	1.98
6	184.35	44.61	21	0.3	0.28	36	1.35	1.36
7	0.13	0.41	22	0.22	0.22	37	0.13	0.15
8	2.53	1.77	23	0.41	0.41	38	17.15	16.88
9	6.78	3.3	24	5.22	5.23	39	8.36	8.32
10	0.22	0.51	25	0.07	0.07	40	2.84	2.63
11	19.02	18.93	26	0.33	0.33	41	1.05	1.04
12	0.91	0.61	27	1.18	1.1	42	0.85	0.82
13	56.64	19.09	28	2.36	2.35			
14	1.8	1.74	29	9.87	9.83			
15	26.61	15.03	30	1.95	1.92			

## Discussion

Accurate detection and monitoring of glaucoma progression are cornerstone for a better patient care and making appropriate clinical decisions. Trend analysis of longitudinally collected perimetry data remains one of the most commonly used approaches for monitoring progression of visual field in glaucoma patients. Despite the availability of many advanced approaches for tracking visual field progression over time, OLSR has still been used for fitting VF data from an individual eye. Repeatedly measured MD values are inherently sequential and are potentially correlated. For example, MD value at one time point is often correlated with previous values. Data independency, a core assumption of OLSR method is often violated by MD series. The OLSR approach ignores the correlated error resulting overestimation of the significance of the trend. As discussed in Pathak et al.^10^, if OLSR is imposed upon data that are accelerating downwards exponentially, the residuals will tend to be positive in the center of the series, and negative at early and late visits in the series. Thus, a linear model, being a poor description of the mode of change occurring within the series, causes the appearance of significant temporal autocorrelation. Additionally, the OLSR is based on the assumption that data is normally distributed. This assumption is often compromised for MD series from an eye. To sum, forecast made using OSLR are mere extrapolation of the observed linear association between MD and covariate time. Extrapolation does not account temporal dependencies, a crucial component of longitudinal data for making forecast at the future time.

Several time series methods are available to fit temporally correlated data among which AR (1) could be an option to fit MD series. However, the use of AR (1) to fit MD series is also severely restricted. This is because AR (1) and other time series methods assumes equally-spaced measurements and normally distributed data. Patients’ visits to doctor office however are generally irregularly-spaced and MD data do not always follow normality. The IAR (1) method is specially developed to model irregularly-spaced time series data. Another advantage of using the IAR (1) method to fit MD series is that it can be used to fit both Gaussian and non-Gaussian errors^24,25^. This is particularly important as MD data often do not follows normal distribution. On the other hand, IAR (1) as a time series model, incorporates correlated data and lagged dependency among the MD data. Thus, compared with OLSR fit, the IAR (1) method markedly improves the validity of the model as well as provides better forecasts than OLSR.

Both approaches used in this study have some limitations. We only include MD series with twenty-five or more observations. This is because our main goal for this study is to see if IAR (1) is statistically more valid approach and offers better fit and forecast than OLSR. A relatively longer series allows us to put aside some observations as a test data. Furthermore, only MD series that exhibits significant temporal correlation or deteriorates significantly over time are included. This limits the wider application of IAR (1) across all spectrum of disease progression. Including eyes with shorter series may bring more insights of overall applications of the IAR (1) methods. Furthermore, finding minimum series length for getting stable estimates of the model parameters as well as finding a breaking point where IAR (1) seems consistently better than the OLSR are worthy to explore.

The IAR (1) is more valid than an OLSR analysis, but it is also not optimal. Like other regular time series models, it is a statistical method to analyze stationary data, and in its most common formulation assumes homoscedasticity, which is not met with data from perimetry. OLSR and IAR (1) are both linear models, and thus are not optimal to predict asymptotes, such as that caused by the perimetric algorithms censoring data below 0 dB (due to hardware limitations rather than any true physiologic asymptote. One reason for the improved performance of IAR versus OLSR is that interventions, in the form of changes to medications and/or changes in adherence and/or surgery, can cause the true trend to deviate from linearity. IAR does a better job of automatically adjusting for such non-linearities, which manifest as correlated residuals from a linear trend line.

## Conclusions

To conclude, the OLSR is inappropriate to model longitudinally collected MD series as it violates the core assumptions of normality and uncorrelated error. Predictions made using OLSR does not account temporal dependencies between MD values and thus forecasts are biased. Time series methods such as IAR (1) are specifically developed to account temporal dependencies between chronological data. Forecasts made using IAR (1) method incorporates relationship between present to its own values as well as patterns and trend in the data. IAR (1) is also a suitable method to model both normal data as well as data with heavy-tailed distribution such as t-distribution. Considering its better predictive capability and being a statistically more valid method to model correlated errors, Gaussian and non-Gaussian, the IAR (1) model can be used as a better alternative for fitting and forecasting MD series in glaucoma patient. The IAR (1) appears to perform well over OLSR when the MD values are correlated.

## Data Availability

The datasets used and/or analyzed during the current study available from the corresponding author on reasonable request.
